# Practices of Care and Relationship-Building: A Qualitative Analysis of Urban Agriculture’s Impacts on Black People’s Agency and Wellbeing in Philadelphia

**DOI:** 10.3390/ijerph20064831

**Published:** 2023-03-09

**Authors:** Ashley B. Gripper

**Affiliations:** The Ubuntu Center on Racism, Global Movements, and Population Health Equity, Dornsife School of Public Health, Drexel University, Philadelphia, PA 19104, USA; abg66@drexel.edu

**Keywords:** community gardens, urban agriculture, environmental health, spirituality, wellbeing, environmental racism, collective agency, community power, Philadelphia

## Abstract

Gardens and farms provide individuals and communities with access to affordable, nutritious, and culturally significant foods. There is a rich body of literature unpacking the connections between Black urban growing and agency, freedom, resistance, and care. However, spirituality remains one aspect of health and wellbeing that has not been studied extensively in relation to agriculture. The main goal of this study was to conduct focus groups with Philly-based growers to understand the self-determined impacts of urban agriculture on health, agency, and wellbeing. The secondary goal of this work was to determine if these impacts differ by race. I apply a collective agency and community resilience theoretical framework to this study. This framework offers a model to understand agriculture as a way for communities to become self-determined, self-reliant, and self-sustained. For this study exploring the impacts of urban agriculture on health, there were three eligibility criteria. Participants had to be at least 18 years old, identify as Black or White, and have grown food in a garden or farm in Philadelphia. I hosted six race-specific focus groups at Bartram’s Garden in Southwest Philadelphia. The audio recordings were transcribed, and the full transcripts were coded using open and axial coding methods and a “key concepts” framework. We also employed several methods of triangulation to help ensure the credibility and validity of the findings. Four major themes emerged from the data: growing as a demonstration of agency and power, growing as a facilitator of body–mind wellness, community care and relationship-building, and deepened spiritual connection and interdependence. There were both similarities and differences in the impacts of urban agriculture by race. Across the six focus groups, people talked about concepts related to community care and relationship-building as being major benefits of growing food. In both groups, people also brought up significant issues and barriers around land security. Mentions of spirituality appeared more frequently and more emphatically in the Black focus groups. Black focus groups were more likely to discuss the collective impacts of agriculture, while White participants were more likely to discuss the impacts on themselves as individuals. The findings of this focus group study point to some key domains through which agriculture impacts the health of farmers and growers in Philadelphia.

## 1. Introduction

Inadequate access to nutrient-rich, affordable, and culturally significant foods is a form of slow violence [1]. Rob Nixon defines slow violence as “a violence that occurs gradually and out of sight, a violence of delayed destruction that is dispersed across time and space, an attritional violence that is typically not viewed as violence at all” [1]. Access to fresh, chemical-free produce is often harder to find in low-income Black neighborhoods than more affluent or White neighborhoods [2,3,4,5,6,7]. When Black communities have access to supermarkets or grocery stores, they generally offer lower quality produce or carry produce that is not affordable to Black communities with lower incomes.

These barriers to nutrient-rich, nourishing food can be described as food apartheid. Food apartheid is different from a food desert. Deserts are naturally occurring landscapes that can be rich with life and food. People who are unfamiliar with deserts often do not know where to look for that food. Food apartheid, on the other hand, highlights the structural factors and actors that have contributed to an intentionally inequitable and inadequate food system for Black Americans and other groups. Food apartheid, food insecurity, and the extraction of resources from Black communities has led to disturbingly high rates of disease and mortality among Black Americans [2,6,8,9,10,11,12,13,14].

In Public Health research, Black people and Black communities are often characterized through a “damaged-centered” research lens and by the disparities and inequities they experience in comparison to White people [15]. While studying health inequities offers incredible value to naming the problems that people face (which is necessary to create helpful interventions), deficit framing should not be the only analysis used to characterize Black people and Black communities. In response to food apartheid, Black people across the country have engaged in acts of resistance to heal and care for themselves, especially when public health interventions have been ineffective at meeting individual and community needs [16,17,18,19,20,21,22].

Over the past several decades, the presence of urban agriculture has rapidly increased in cities across the United States [23,24]. Due to the fact of food shortages during the COVID-19 pandemic, many people are realizing the benefits of urban food-growing for themselves [25,26,27]. In the early days of the pandemic, people flocked to grocery stores to ensure they had enough supplies to get through lockdowns. This led to empty food shelves and limited nutritious options, especially for people in low-income areas. Particularly for Black Americans, the COVID-19 pandemic restrictions and protocols have allowed space and time for many people to participate in gardens, farms, and urban growing practices.

Gardens and farms provide individuals and communities with access to affordable, nutritious, and culturally significant foods. They offer exposure to greenness, opportunities for physical activity, and potential benefits to the microbiome [28,29]. These spaces often facilitate spiritual connections to land. They bolster climate change mitigation and ecosystem services through stormwater retention and cooling effects for urban heat islands [30,31,32,33]. Community growing spaces facilitate information exchange and intergenerational knowledge sharing [17]. They are a means of strengthening social capital and support [18,34] and offer community members spaces to gather and celebrate without fear of crime or state-sanctioned violence. Agricultural spaces led by people of the global majority (PGM) [35] demonstrate the capacity of a community to engage in shared decision-making and community-based solutions to health inequities [17,18,19,36,37]. PGM refers to people of the global majority. This includes Black, Indigenous, and Asian people and other people of color. Originators say, “this wording points out the demographic inaccuracy of the euphemism ‘minority’ and can feel more empowering for some people”.

Dyg et al. (2020) performed a thematic review of the literature on community gardens and health [38]. While community gardens can be a form of urban agriculture, not all types of urban agriculture are community gardens. This review identified a total 51 studies that investigated the relationship between community gardens and health. The studies were quantitative, qualitative, and employed mixed methods. Common designs included case studies, cross-sections, and interventions. Overall, this review found that community gardens are consistently positively associated with relationship-building and social connection. The associations between community gardens and other forms of health were mixed, although many studies showed positive impacts on physical health, food knowledge, and communities/neighborhoods [38].

Other urban agriculture and health literature has demonstrated positive associations between agriculture and mental health [34,39], specifically depression and anxiety [40,41]. However, there is an increasing body of literature that provides evidence that the leadership of food-growing space also matters [19,42,43]. There may be a difference in the impacts of urban, community food-growing depending on if a project is community-led or led by people from outside the community [42,43,44]. For instance, although White-led urban agriculture projects situated in communities of color tend to receive more funding than PGM-led projects [35,45], their programming has weaker impacts on the communities they serve [46]. In a literature review on the impacts of urban agriculture, Dr. Sheila Golden found that the “culture around local and healthy food has often been associated with those that have higher-educations and incomes,” and that this type of urban agriculture can be exclusionary to low-income Black communities, who are often the target of urban agriculture projects [42,43,44].

In addition to increasing access to nutritious foods, PGM-led spaces offer additional community benefits. These spaces foster community connection, agency, and resistance. For example, through two qualitative studies on farmers in Detroit, Dr. Monica White demonstrated how Black-led community food-growing projects do more than provide fresh fruits and vegetables [17,18]. They help foster community self-determination, self-reliance, and activism [47,48]. Other qualitative literature has also shown that urban, community food-growing positively impacts the physical health, community development, and social capital for those who engage in it [34,43,49].

There is also a rich body of literature unpacking the connections between Black urban growing and agency, freedom, resistance, and care. Scholars in anthropology and sociology have used case studies to show that agriculture fosters power and collective agency within Black communities [17,18,20,21,37]. A major way that Black communities, particularly agricultural communities, organize is through creating access to resources that have historically and socially been denied to them [36].

Spirituality is one aspect of health and wellbeing that has not been studied extensively in relation to agriculture. Afro-indigenous people have kept and expanded agricultural knowledge and techniques for tens of thousands of years. European colonizers kidnapped Africans from their continent and enslaved them in a foreign land to capitalize off their agricultural knowledge and wisdom [50]. The atrocities committed—genocide of people Indigenous to the Americas and the brutal enslavement of people Indigenous to Africa—were conducted so on this land and for the theft and commodification of this land.

In his book *Something Torn and New*, Ngugi wa Thiong’o talks about the dismembering of the African continent and people [51]. He says, “The requirements of the slave plantation demanded the physical removal of human resources from the continent to work on land stolen from other peoples, mainly native Caribbeans and Native Americans. The result was an additional dismemberment of the diasporic African, who was now separated not only from his continent and his labor but also from his very sovereign being”.

Dismembering serves a specific purpose. It seeks to separate people from their spiritual practices, from their foodways, and from their relatives both human and more-than-human [52]. The goal of dismembering is humiliation, and it is also to prevent re-membering [51]. When Black people forget who we are, where we come from, and our ways of knowing and loving and living, then it is easier to control what we eat and control us. I use “we” because I am a Black woman working to help other Black people reconnect and rebuild our relationships to our ancestral foodways, spiritual practices, land practices, and culture.

Settler-colonizers attempted to dismember Africans from their cultures, rituals, spiritual practices, languages, and foodways while extracting their ancestral farming knowledge. Unbeknownst to them, abducted African women often braided seeds from home into their hair, carrying with them their stories and power across oceans. Though in many ways the goal was dehumanization, dismemberment, and disconnection, Africans preserved and maintained their spiritual practices and ways of relating to each other and to Earth.

In “Growing Black food on sacred land: Using Black liberation theology to imagine an alternative Black agrarian future,” Dr. Priscilla McCutcheon uses Black liberation theology to understand the presence and role of “Spirit” in Black farming communities [53]. In her work, McCutcheon argues that spirituality should be an important factor of consideration for researchers studying Black farming and agrarian communities. To date, there has been little scholarship exploring the relationship between spirituality and agriculture [17,53,54].

There are many definitions for “urban agriculture”. I define urban agriculture as the process of growing food outdoors in a city or urban area. My work and this study explore the impacts of land-based urban agriculture on health, spirituality, and communities. I consider urban farmers and gardeners to be anyone who grows food on a farm or in a garden, and in a city, respectively. While the USDA has stricter guidelines concerning what qualifies someone to be a farmer [55], I choose to adopt an approach that includes and values the labor of all people who tend to and grow food on land of considerable size. This definition of urban agriculture and urban farmers is consistent with what others have outlined in sociological literature [36].

### 1.1. Theoretical Framework: Collective Agency and Community Resilience (CACR)

Collective agency and community resilience are widely practiced within Black agricultural communities [18,20,21,56]. In her book entitled *Freedom Farmers: Agricultural Resistance and the Black Freedom Movement*, Dr. Monica White describes the theoretical framework of collective agency and community resilience (CACR) as capturing “the activities communities enact as a means to be self-reliant and self-sufficient ” [36].

This framework offers three primary strategies through which agriculture helps Black communities realize their collective agency and resilience. The strategies are commons as praxis, prefigurative politics, and economic autonomy. Commons as praxis refers to the resource commons and includes collective decision making, collective action, and fair allocation of shared resources. Prefigurative politics involves a process of creating and building alternative democratic political systems, particularly when the dominant political system excludes Black people, Black ideas, and Black interests. Economic autonomy refers to “efforts to create an alternative system of resource exchange within the community, and these funds and resources have direct benefits for all of its members” [36]. Fostering economic autonomy can include the development of economic systems alternative to capitalism, such as socialism, or more cooperative economic practices. Bartering and labor exchange practices can also help increase economic autonomy for Black farmers and growers.

As a framework, collective agency and community resilience highlights and unpacks the role of agriculture in helping Black communities become self-determined, self-sustained, and self-reliant. I use this framework as a guide to understanding how participation in urban agriculture primarily impacts Black growers in Philly and their communities. This study also looks at the impacts on White urban growers primarily as a comparator.

### 1.2. Collective Agency

White describes collective agency as involving “social actors’ ability to create and enact behavioral options necessary to affect their political future” [36]. She goes on to argue “a community does not have consciousness in the same way an individual does, but when a group of people comes together and believes in their mutual success, this creates a separate type of consciousness that drives collective agency” [36]. Collective agency, as I apply it in this study, is not limited to the behaviors that affect political futures but also includes behaviors that affect holistic community healing and wellbeing. It refers to the ability of a community to work together to understand, realize, and act on a course of action to support their political power, wellbeing, and collective self-determination.

### 1.3. Constructive Resistance

White’s theoretical framework on collective agency and community resilience also highlights the ways farmers engage in constructive resistance. She compares constructive resistance to Benedict Kerkvliet and James Scott’s concept of “everyday strategies of resistance” [57]. Everyday strategies of resistance describe how individuals engage in small, disruptive actions to highlight their opposition to injustice, while White’s theory focuses on the constructive actions that Black farming communities take to resist oppressive social, political, and economic conditions [36]. Often, these constructive forms of resistance result in improved psychological, physical, spiritual, and social health for communities oppressed by settler governments and regimes.

The primary aim of this study was to conduct an exploratory focus group study with Philadelphia-based growers. The goal was to understand the self-determined impacts of urban agriculture on health, agency, and spiritual wellbeing. The secondary goal of this work was to determine if these impacts differed by race. Using a mixed-methods approach, the findings from this body of work will be used to inform the development of a scale that assesses how spirituality, wellbeing, agency, and power manifest in urban agriculture communities.

## 2. Methods and Analysis

### 2.1. Research Design Overview

Being deeply familiar with the study population, I recognized that richer data would come from having growers in the same room listening to, learning from, and in conversation with other growers. I have worked closely with the study host site, Bartram’s Garden, as well as Philadelphia-area urban agriculture organizations for several years helping to design, host, and implement community programs and events. These carefully cultivated relationships allowed me access to the stories and experiences of communities of farmers and growers in Philadelphia.

I chose a double-layer design because there are two categories of experiences that this research aims to capture [58]: Black urban growers and White urban growers. Black growers are the primary population of interest and make up one of the largest demographics of growers in Philadelphia. Most urban growers in Philadelphia are Black, White, Latino/a/x, or Southeast Asian. While this study focuses only on the experiences of Black and White growers, future work will include the experiences of Black, Indigenous, and other growers of color in Philadelphia and other cities.

To allow for greater expression of thought and experiences, focus groups were organized by racial group. I chose focus group methodology to allow for the natural conversation that would emerge between urban growers. Focus groups are generally the preferred method when the goal of research is to explore people’s thoughts, beliefs, and attitudes toward a particular issue or subject [58]. This method is useful when one wants to understand how perspectives may differ between different groups of people or across geographic regions. Focus groups are also advantageous when “the researchers need information to design a large-scale quantitative study” [58]. The information and insights that were gained through this focus group study were directly translated into a scale for related research.

Data often emerge “through interaction within the group” [58,59]. Particularly among African Americans, oral and linguistic traditions, such as call-and-response and “co-signing”, are a big part of the culture [60]. Call-and-response, traditionally practiced in African American church, music, and theater, involves audience participation—responses—to the main actor’s prompt—call. Within these focus groups, I wanted to capture the ways that Black participants might engage in call-and-response by responding to each other’s prompts and co-signing each other’s statements. Considering that one of my goals is to understand collective and community processes of agency and resistance, then it is appropriate to use a conversational method, such as focus groups, which will allow those characteristics to emerge.

### 2.2. Recruitment Process

For this study, there were three screening/eligibility criteria. Participants had to be at least 18 years old, identify as Black or White, and have grown food in a garden or farm in Philadelphia.

For each category of participant, it is an acceptable practice to have at least three focus groups so that we reach the point of saturation in our data collection—that is, are we still learning new information from these groups, or, are the same key concepts and themes being repeated [58,61]? Since there were two categories of participants, we hosted six focus groups with five to eight participants in each group. Groups with fewer than five people have the potential to lack variation, while groups with more than eight people may be difficult to manage. Five to eight people was the compromise between variation, saturation, and feasibility. My target goal for enrollment was 30–48 people.

### 2.3. Participant Selection

Participants were identified through snowball sampling. I leveraged key community partners and influencers in Philadelphia’s agriculture communities to help with recruitment (see table in appendix). Participants were also recruited through several social networks and urban farmer listservs. Since urban farmers and gardeners are a niche group in Philadelphia, the most appropriate recruitment strategy was to rely heavily on the networks of grassroots and nonprofit community partners. Recruitment goals were met two weeks after outreach began. All eligible participants were enrolled in the study (*n* = 42), but some had to withdrawal prior to the focus groups due to schedule conflicts and inclement weather (*n* = 10). Across six focus groups, we engaged 32 people. Twenty participants racially identified as Black, and twelve participants racially identified as White. All participants completed a screening questionnaire prior to enrollment, COVID screening, and demographic questionnaire.

### 2.4. Data Collection

All data were collected through focus group interviews and questionnaires. I obtained demographic information through electronic and paper surveys. The assistant moderator, and I conducted six focus groups from August 2021 to September 2021 at Bartram’s Garden in Southwest Philadelphia. They were split evenly by race, so there were three White and three Black focus groups. The groups were staggered between evenings and weekends to accommodate a variety of work schedules. Each focus group ranged from 80 to 110 min, with an average interview time of 90 min. After brief introductions and an overview of the study, we asked permission from the participants before beginning the audio recording. Throughout the focus groups, the participants were asked six primary questions: (1) Why do you farm or grow food? What does it mean to you? (2) Do you think being a farmer or grower impacts your health? If so, how? (3) Do you think urban agriculture impacts your community? How? (4) What are the benefits and/or barriers to farming or growing? (5) What strategies have you used to respond to these barriers? (6) Is there anything else you would like to share about your experiences with urban agriculture?

I asked probing questions to gain further clarity on some of the concepts, ideas, and experiences shared. Participants were encouraged to talk directly to others and respond to or build off statements made by other members of the focus group. During focus groups, I took notes on key themes and ideas that emerged, as well as my own thoughts and responses regarding the content of group conversations. The assistant moderator was responsible for managing the audio equipment and taking notes on body language throughout the focus groups. At the conclusion of each focus group, I asked participants to provide feedback on the moderation and flow of the focus group. If comfortable, they were given the opportunity to share verbally in a communal setting. The participants were also given electronic or paper follow-up surveys to gauge their feedback on the focus groups. Each participant who completed a focus group was compensated at a competitive rate of USD 100 as a symbol of appreciation and respect for the time and energy they had given to this research.

In the first three interviews, I asked participants to write down important areas of consideration for further urban agriculture research at the end of the focus group. Realizing that it made more sense to have people write down their ideas in the beginning of the focus group, I shifted to asking participants to write down topic areas before the focus group dialogue began. In the early focus groups, I asked fewer probing questions and was much more “to the point”. As I became more comfortable and rejected the mythological notions of researcher objectivity and professionalism, I leaned into being more of my authentic self. This led to asking more probing, sometimes challenging, questions of participants. The conversation seemed much richer as a result. The response from participants was also affirming of this approach.

### 2.5. Analysis

To understand the impacts of urban agriculture on the health of growers, this study used several methods of analysis. We conducted a transcription-based analysis, which means that entire focus group audio recordings were transcribed. I employed a “key concepts” framework. This framework was a good fit for this study, because it focuses on discovering core ideas and understanding how participants conceptualize and relate to a topic. A key task of this framework is to “identify a limited number of important ideas, experiences, or preferences that illuminate the study” [58]. The analyses of the transcripts were computer-based and performed in both NVivo and Taguette, while still employing many elements of the classic approach. I compiled transcripts by category of participant. One section included all of the transcripts from the focus groups with Black growers in Philadelphia and another section included all to the transcripts from White growers in Philadelphia.

I compiled a list of preliminary codes (~34) based on the notes from the focus groups, memory, CACR theoretical framework, and my lived experience as a Black urban grower. Using the preliminary codes, the assistant moderator and I independently performed a combination of open and axial coding. The final codes represented a combination of preliminary codes and codes that emerged during the transcript-based coding process. After we completed the initial coding, we met to compare codes, highlight differences, and arrive at a consensus regarding the findings. Once the coding was complete, I organized the codes into major themes.

I also employed several methods of triangulation to help ensure the credibility and validity of the findings. In addition to multiple coders, I coded and recoded transcripts multiple times, conducted focus group debriefs, engaged in continuous processes of self-reflection, performed “member checks,” and carefully reviewed the follow-up survey responses [59,62,63,64].

Overall, the analysis process was continuous and reflexive. The insights and findings for the impacts of agriculture on health are described in the next section.

## 3. Findings

The findings show that urban growers in Philadelphia have a range of experiences when it comes to agriculture’s impacts on health and wellbeing. These impacts are both similar and different by race. Using an interpretivist approach—accepting that there is not only one but multiple ways and perceptions of how people’s health can be shaped by agriculture –we identified 89 total codes that I then organized into seven themes. The findings section will concentrate on the four most prominent themes: growing as a demonstration of agency and power, growing as a facilitator of body–mind wellness, community care and relationship-building, and deepened spiritual connection and interdependence. Some of these themes were more pronounced based on the racial makeup of a focus group. The other themes that emerged, to a much lesser degree, are environmental health knowledge, perceptions of self, and barriers and strategies to growing food. The findings in this section are organized by prominence of the theme. Within each thematic section, the data are accompanied by interpretations of quotes and, at times, organized by race. To protect the confidentiality of the participants, aliases are used for the focus group participants throughout the remainder of this article.

### 3.1. Community Care and Relationship-Building

The concept of community care and relationship-building was the top theme that emerged across all six focus groups. I have been interrogating how this theme differs from the well-known concept of social capital and have several conclusions. Social capital, by name, arises from the concept of capital and capitalism [65]. Its first articulation in academia is commonly attributed to Pierre Bourdieu, James Coleman, and Robert Putnam [65,66]. It focuses on the resource—the *what*—that is being shared through a network [65].

The concept of “community care” and the value placed on “relationship-building” have roots in grassroots organizing and activism [67,68]. Civil rights movements and organizing practices prioritized relationships and care for one another along with their political goals [68]. Community care and relationship-building as a theme places emphasis on the people *for whom* resources are being exchanged. These concepts uplift the care and nurturing of one another. Community care and relationship-building are not about what is being shared but *why* it’s being shared. The focus here is on the people for whom resources and knowledge are being distributed.

People in both the Black and White focus groups discussed community care and relationship-building in the context of preserving and restoring familial relationships and bonds. Julie, a participant in one of the White focus groups, shared that growing food allows her to connect with family members across social and political differences. She said, “It’s helped give me some common ground with my family who I do not always see eye to eye with”. For Megan, a participant in the White focus group, growing food helped her to connect with her family in a slightly different way:

*I feel like it’s one of the only things that people in my family…will come to me for advice about, even though I studied science for a long time, and I worked in neuroscience and literally grew brain cells, and I grew brains in a dish. Like nobody really respects that, but people want to hear how to keep their tomatoes alive, like that’s cool. I like feeling respected.*

In her case, gardening may have been a more accessible and interesting topic to discuss for her family members. Her agricultural knowledge is valued and sought out by her family members and that leaves her feeling respected. For both Megan and Julie, agriculture helped them to connect with and restore familial relationships.

The preservation and restoration of familial relationships had similar undertones in Black focus groups but seemed to be more focused on preserving and passing down agricultural knowledge and wisdom. Dr. Williams described the importance of preserving family foodways and reconnecting with relatives who live in the South. For her, growing food and preserving foodways provided a way for her to pass down her food and family’s traditions to her children.

*As my daughters…start to grow up, I always found myself sharing more recipes. We cook everything from Southern food to gourmet food. My dad was a chef, so I feel the best of both worlds. I moved away but am beginning to share more about some of the Southern food recipes and things that I remember…my mom died in ‘16. And they used to spend a lot of time with her. So now they’re young ladies…and they’ve spent more time going back down to what I call The Family Plot. There’s relatives that live in like six houses in the area. So really getting to know their cousins and things like that and being curious about history.*

Sharing foodways and agricultural history has also provided Dr. Williams’ children with opportunities to build and deepen relationships with their family, especially after the death of their grandmother. Building relationships with and extending care to family is something Kisha also mentioned. She explained that her journey of growing food inspired her family members to be more intentional and active in caring for their mental and physical health:

*I think the reason why I grow food is because I believe that it helps your mental and it helps your body and once you start healing yourself, your family slowly follows you. Because they see, “Oh, like, I’ve seen this person down and off, but now they’re healing,” and you just see flowers as they walk past. Now I’m starting to see my family slowly but surely get in on that path. So I think I farm for my future self and for generations to come in my lineage. So that’s why I do it.*

Her comment also highlights a consideration that Dr. Williams shared—future generations. Both women think of agriculture and their work as helping to preserve agricultural knowledge and pass it on to the next generation. Within the theme of community care and relationship-building, this subtheme is “intergenerational transfer of knowledge”.

While both White and Black participants talked about strengthening familial relationships, the intergenerational aspect only appeared in conversations among Black participants. Another difference that emerged between Black participants and White participants was the way they described agriculture’s impacts on relationship-building.

Within the White focus groups, participants often explained how growing food has helped increase their social connections. Social connection, as it appeared in the focus groups, was about the ways in which agriculture facilitated opportunities to meet, connect with, and learn about other people. Tom shared how growing food has helped him diversify his network.

*And being on the land…men are able to have conversations and…work next to somebody who’s coming from a different background, eat food, share stories. I feel like there are very few other things in this world that can hold the container for that. And I also want to be part of like, I selfishly love doing it.*

Allison and Mary echoed what Tom said:

*Gardening is this intersection of communities in a way that most other places aren’t so intergenerational, interracial, inter socio-economic, I mean, I feel like I have friends and colleagues and deep long like 30-year long relationships with people who I never would come across, or know if it wasn’t for my, my life as a gardener, as a garden educator, as a random volunteer, at a random thing.*

(Allison)

*I want to second, what you were saying that you quickly get into community with people that you wouldn’t have an opportunity to be in community with without the garden connection. And I really value that. And I really like meeting people I wouldn’t meet in my little pass that I go through, and at the garden, it’s a common denominator.*

(Mary)

This idea of gardening helping one to diversify the people with whom they come into contact only appeared in White focus groups. There was no mention of racially diversifying one’s network or relationships in the Black focus groups. While some people in the Black focus groups did talk about the importance of social connection, people more frequently described relationships as a byproduct of community-building and community care.

Community-building is about more than social connection. While community-building certainly encompasses social connection, it often consists of more than two people. It involves processes of community care, healing, and collective action. Building community requires intention and a level of reciprocity not necessary for social connection. Social connections can occur on the surface of interactions, whereas community-building, particularly among Black agricultural people, often involves considerable depth and substance to establish that trust within a group. Often, community-building is what enables the collective agency and collective action needed for organizing and activism. Ms. Crystal, an elder in the Black focus group, explains how the garden can be a space of relationship-building, organizing, and collective action:

*It’s a way of meeting and greeting people and also getting back to the development and preserving of space. It’s a good way to connect with people that you can work with. You got to have folks to join you in the fight or whatever it is that you need. So we get to share, build that community around gardening.*

Ms. Crystal and others in the Black focus groups saw themselves not necessarily as individuals but as part of a larger whole. Tasha explicitly states how growing food has strengthened their identity:

*It’s made me have such a stronger sense of self and identity and connection. And understanding that, like, myself isn’t just me, it’s not an individual. I’m in this community, myself, and the work that I do doesn’t just belong to me, it belongs to my community.*

Seeing oneself as “we” instead of “I” is rooted in Indigenous African traditions and ways of being [69]. This ideology was consistent across the various Black focus groups. It was not as prevalent in the White focus groups. The concept of “othering” was more likely to appear in White focus groups. White participants often seemed to not see themselves as part of the community they were working in, in search of social connection, whereas Black participants tended to talk about themselves as a part of the community. Black focus groups also more frequently discussed the collective impacts of agriculture, while White participants were more likely to discuss the impacts on themselves as individuals.

The theme of community care and relationship-building, as it appeared in the data, also seemed to be reflected in how participants interacted with and treated the environment. There were threads and codes related to climate change and sustainability that emerged. Participants often said caring for plants helped model how to care for and build relationships with people—bringing to life the saying, “as with the land, is with the people”.

### 3.2. Growing Food Facilitates Body–Mind Wellness

Mental and physical wellness were strong themes across Black and White focus groups. Across all focus groups, participants talked about their mental wellbeing in relation to COVID-19 restrictions and protocols. For instance, Ms. Judy, an elder participant in the Black focus groups, talked about her experience quarantining in the garden:

*The gardening, especially this year, and last year, I’d say more so I was not quarantined. I was in that garden. That garden saved me. It helped me with my sanity because I got out there. That was my escape.*

Allison, a participant in the White focus group, also shared that caring for her garden helped her to feel a sense of purpose during the pandemic:

*I think especially during the pandemic, mental health was definitely on an all-time low. There wasn’t much to do, but like going out and tending to my garden gave me a real sense of purpose.*

Gardening and farming seemed to offer Black and White participants opportunities to relieve stress, anxiety, and depression during the COVID-19 pandemic. However, grief processing and grief healing were topics that emerged more often in the Black focus groups than in the White focus groups. Sister Sahar shared how growing food and caring for gardens helped her process the passing of her mom:

*My mom passed away when I was seven. My mom loved sunflowers. So it’s like whenever I look at sunflowers or if I’m going through something, when I was going through something, I came outside and all the dragonflies was flying around—the energy. It’s like my mom. I think about my mom…I don’t know how that happened in my life. Something happened to my life…whereas I was able to connect the dragonfly with my mom. When I’m going through something, I’ll start talking to her next thing you know, here comes a dragonfly and here comes the sunflowers.*

Other participants talked more generally about the impacts gardening or farming has on their mental health. Growing food and having your hands in the soil may impact both mental and physical wellbeing, in part, through soil microbes [28]. Quinn, a participant in one of the White focus groups, shared that working in the soil was the most impactful treatment for their mental health:

*I have been in therapy for a lot of my life. I tried different medications for mental health issues. And some things helped at different points in my life, but nothing was impactful for my mental health as much as being outside and working in the soil.*

Many focus group participants also described agriculture’s impacts on their physical health. People shared a range of reasons as to why they began growing. Many were related to sickness caused by eating processed or junk foods. Ms. Cheryl’s agricultural journey began with pursuing physical wellbeing, but she also shared how her personal journey led to physical wellness for her children as well:

*…but like the other ingredients that have a whole lot of different chemicals in there. And I needed, I wanted to give my organs a break. I wanted time to detox and to heal, rejuvenate, regenerate, whatever it was that I could do to help myself in small steps. And then I got my children into that. So that we all started taking classes as a family. And of course, I was out there a bit more aggressive because I really wanted to begin to control what I was using for my personal healing, and for my children as well.*

Body–Mind wellness seemed to have more similarities across races than differences. Many participants started gardening and/or farming because they wanted to have more control—agency—over their physical health. Especially during the pandemic, people sought being in the garden for the respite, peace, and stress relief that it offered. As articulated by several focus group participants, growing food also provides one with a heightened somatic sensory experience, helping them to realize the deep connections between mind and body.

### 3.3. Land-Based Spirituality and Interdependence

Growing food and caring for the land have also helped people to nurture greater spiritual wellness and practices. Through being on the land, people have developed deeper connections to Earth, to more-than-human beings [52], to each other, and for some to a higher power and their ancestors.

For Black and White participants, a primary way that agriculture has helped facilitate a deeper spirituality is through connection to Earth. Within White focus groups, this was most often through interdependence or Ubuntu. Ubuntu is a Zulu proverb that means “I am because we are” or “I am a person through you” [70]. Ubuntu is about the interdependence and interconnection of all humans. This quote from Sarah, a participant in one of the White focus groups, demonstrates a realization of interdependence:

*I guess I always had this relationship with other species or other life forms and working with them and building relations with them. And I don’t think it’s all that different with plants. I think just working with other living things that aren’t humans. I was thinking about that when you guys were talking about making mistakes because it’s like not all entirely in your hands. Like even if you plant too many, tomatoes are still going to produce tomatoes, maybe not like optimal amounts, but I like things like that. It’s like a collaboration. And I think that that makes me more aware of like more of a collective existence and less of an individualist like getting caught up in myself, in my own problems and what I’m experiencing more like, witnessing, and collaborating with other species. Yeah, it makes me feel less isolated, I guess.*

Sarah uses words and phrases such as “collective existence” and “collaboration” to illustrate how growing food has helped her to understand and think of the interdependence of all inhabitants of Earth.

Ubuntu/interdependence was also a subtheme of land-based spirituality that appeared within the Black focus groups. Mostly, connections to Earth seemed to be more often described in these groups as talking with and hearing from plants. Kisha shared what the experience was like for her in her beginning days of farming:

*When I first started farming, I actually didn’t want nobody to know that I was farming. And I say that to say, because, like, the things that the plants were telling me, it just seemed like it’s something, the things that I was learning in that process, it just seemed like it was something that was just so sacred. Like, it felt like, I don’t really want to use this word, but I’m going to use it felt like alchemy. It felt like I was doing magic with the soil by just knowing that I can plant a seed and it could come up.*

She uses words like “sacred” and “alchemy” to refer to the relationships that she was developing with plants. Other participants in the Black focus groups expressed similar sentiments to Kisha. Some described their relationship with plants as being ancestral. Tasha broke down the ways farming has helped them reconnect with their ancestors and reclaim their ancestral agricultural knowledge:

*I realized that like, I don’t think I knew who I was until I started doing this work. And there’s both this like mental health and spiritual component. Because doing this work, like I mentioned earlier, I feel like I found my ancestors, and there’s this undeniable connection and communication that happens. That has just given me like this sense of belonging and knowing. And as soon as I started farming, it just felt like, this is where I’m supposed to belong. This is what I’m supposed to be doing. And there’s this joy, this absolute joy that this work brings me. Part of it is like the symbiotic connection of just being on the land. And doing that work and being part of the rituals of weeding and hoeing and prepping rows and planting. And then also being in community, and just getting to meet so many people that are about this work, or who want to be about this work.*

Tasha offered valuable contributions to the conversation by naming the “symbiotic connection of just being on the land” and the “rituals of weeding and hoeing and prepping rows and planting”. Several of the Black growers talked about agriculture’s impacts on their spirituality through participating in rituals. Many of these rituals were actions, prayers, and ceremonies performed by their ancestors, who were also farmers and growers. In addition to rituals, some participants talked about the garden, and particularly soil, as a facilitator of deeper spiritual connections. Sister Sahar shares:

*The secret Is in the garden and people just don’t understand. You just literally have to go in the garden to visit God. And God just starts talking to you and you just start connecting with the soil and your spirit is being cleansed by just touching the soil and that energy…is just like going into the soil and coming back up your legs. And when you walk out, it’s like, I’m in power.*

Sometimes these experiences can feel unique to Black people. Kisha offered the following response to another focus group participant who shared an experience connecting to her husband when he became an ancestor. Kisha responded to this participant saying:

*I was getting chills hearing about it, because sometimes I feel like you will be having all of these things happening that I feel like only Black people can understand. It’s like a spiritual, it’s a rooted experience. Like, what you’re saying people will be like, “Nah, that’s something that you hear on a movie”. But like my people really be going through these real lived experiences that you just can’t explain.*

Within the Black focus groups, spirituality was largely captured through connection. Connection was described in relation to God or a higher power, to ancestors, and to Earth. That connection was realized through ritualized actions, such as weeding, planting, and hoeing. These connections were also realized through simply being present in a garden or on a farm and being open to receiving communication from plants and all the life around. In the White focus groups, spirituality was a far less common topic of conversation; however, when it was, the participants described feeling connected to Earth and understanding the interdependence of everyone on this planet.

### 3.4. Growing Food as a Demonstration of Agency and Power

Patricia Hill-Collins defines agency as “an individual or social group’s will to be self-defining and self-determining” [71]. I consider power to be the actualization of agency. In the context of urban agriculture, growing can be a demonstration of agency and power because of the ways it allows growers to be self-defining and self-determining. Across focus groups, but more prominently in the Black focus groups, agency and power were conveyed through conversations about economic alternatives to capitalism that enhance community wellbeing, as well as the importance of financial security. There were conversations about self-reliance and self-sufficiency, sovereignty, negative impacts of capitalism, and the process of unlearning. In many of these conversations, the participants named how agriculture has facilitated a process of re-educating themselves about food, economic systems, resource allocation, financial stability, and political systems. Many participants also talked about how they practice and actualize what they have learned from growing food with others. They spoke of unlearning incomplete truths that uphold the status quos of society. These incomplete truths were often in reference to societal norms, expectations, or lessons taught in school. The underlying theme that connected these threads is growing as a demonstration of agency and power.

Truly knowing and understanding what you eat requires knowledge of where your food comes from and how it was produced. Instead of relying on someone else to tell you where food comes, you can rely on yourself because you grew the food. This is providing agency with respect to food. Being able to actualize this agency is power. Jada offered one example of how agency is demonstrated in the context of agriculture. In the Black focus group, she explained how growing food provides agency with respect to one’s health:

*For me, growing food, I talked a little bit about this, it gives you some agency over your health. And to me…that’s powerful and the work that I do, and the conversations that I have with folks in the community…It feels like, I can make these decisions. And this is something that I can control, whenever we think about our health and what happens, what’s going on in our bodies, there’s a lot of things that’s out of our depth of knowledge. And so it encourages you to, like, seek.*

Similarly, Ms. Valerie spoke to the importance of agency and how growing food can help people become more self-sufficient and not rely on city government to bring supermarkets and quality food to Black neighborhoods.

*We need to know how to be self-sufficient. And know how to do for ourselves. So that’s also one of my goals is to teach people about backyard chickens and how to raise them, how to handle things, how to grow, how to plant and plant raised with garden beds. So that’s it for me.*

One way to increase self-reliance is through growing food and raising animals. We can increase community self-reliance by passing on knowledge, wisdom, and skills to other people and generations. As an older woman, Ms. Valerie often highlighted her goal of transferring knowledge intergenerationally and how that facilitates agency and power.

Ms. Crystal and Imani highlighted another aspect of agency and power: resistance. In “Sisters of the Soil: Urban Gardening as Resistance in Detroit,” Dr. Monica White characterizes resistance as being in response to “the social structures that have perpetuated inequality in terms of healthy food access,” and as the ability to “create outdoor, living, learning, and healing spaces for themselves and for members of the community” [17]. For Ms. Crystal and Imani, participating in urban agriculture provides them with the opportunity to organize and strategize on how best to obtain the resources their communities need to thrive.

*And I feel like my strategy for that is to refuse and resist those people as much as possible, and to get on they nerves until they answer the phone. Because I’m done participating and trying to do things in a way that like our White supremacist society prescribes us to do. It isn’t a solution for the issues that we have in our community. We got to move on our own time. So that’s how I feel about it.*

Here, Imani also speaks to the challenges of trying to operate within a White supremacist, or White violent, society. Her solution is to forge a path for her community to care for themselves through gardening and farming. Ms. Crystal also speaks of resistance by using phrases such as “join you in the fight”. She talks about resistance in relation to community organizing while also highlighting agriculture and the garden as a facilitator of community and relationship-building. This quote was referenced earlier to describe sentiments of community-building. It also speaks to agency and power:

*It’s a way of meeting and greeting people. And also getting back to the development and preserving space, it’s a good way to connect with people that you can work with, you got to have folks to join you in the fight or whatever it is that you need. So we get to share, build that community around gardening.*

Participants in the White focus groups, at times, used similar language as participants in the Black focus groups to describe their experiences around agency, power, and resistance. Two examples of this are from Tom and Quinn:

*But I also, like, want to be part of a larger movement for justice.*

(Tom)

*The first time you plant a seed, and then eat something that comes from it, it’s just like a realization that you could be connected to your food and the earth and in a way that certain systems try to keep you from. So in that way, it feels a lot like resistance to me too.*

(Quinn)

*And I’m really interested in farming and growing one’s own food and food for the community as a form of resistance. Active resistance against forces that try to keep people separate from their ways, their ancestral foodways and their present foodways and try to put as much space between communities and source of food as possible as a way of controlling them and keeping them oppressed. I’m really interested in people learning how to grow food for themselves and their community.*

(Quinn)

Mentions of resistance and justice were coded as examples of activism and resistance. “Activism and resistance” was one of several subthemes that made up the larger theme, “growing food as a demonstration of agency and power”. An interesting thread that appeared across White focus groups was a desire to be a part of a “larger movement for justice”. Some participants talked about how agriculture helps them to feel connected to resistance and justice work for Black and other PGM communities, even though they do not necessarily belong to the communities experiencing multiple systems of oppression.

Quinn, a nonbinary person in their early twenties, exemplifies an understanding of shared language around *agriculture as resistance* but comes from a very different set of experiences than many of the people in the Black focus groups. For instance, Quinn relocated to the Philadelphia area after college and worked as an urban grower while most of the Black participants were native to Philadelphia living in some of the city’s most rapidly gentrifying neighborhoods. They, Quinn, mentions how systems “try to keep you from” realizing your connection to the Earth and food. It raises the question of, to which systems is Quinn referring? When they talk of systems and systems of oppression, does this also include systems of gentrification, displacement, and neighborhood resource extraction?

Resistance is not just about gardening or farming or knowing where one’s food comes from despite the trend we see among White people to claim gardening and farming as resistance. There is a difference between gardening as a hobby and growing food as resistance for yourself, your family, and your community. Growing as resistance, as collective agency, as community power, is about working against the intertwined systems and structures that seek to oppress communities, particularly Black and PGM communities in America. Growing food can be a model of constructive resistance and collective agency but not when it is focused on the individual benefits of growing food for a single person who already holds immense privilege; or when it involves a person of immense privilege entering Black communities hoping to help or save those communities. Growing food as constructive resistance means “the aggrieved actively build alternatives to existing political and economic relationships” [36]. It is how people living under oppression engage in radical acts of “building knowledge, skills, community, and economic independence” [36].

Some participants in the White focus groups were quicker than others to acknowledge the privilege that they have and how growing food is different for them than it may be for someone else. One example of this is from Megan. She says, “I feel like there’s a, I mean, a part of my own privilege where I’m not reliant on the food that I’m growing”.

As participants in the White focus groups engaged with ideas of self-reliance and self-sufficiency, another concept that emerged was around future preparedness or “dooms-day prepping” [72]. This relates to the theme of growing food as a demonstration of agency and power because participants talked about growing food as a skill necessary for taking care of oneself and surviving in the future. Both Quinn and Danielle referenced this skill in relation to Octavia Butler novels:

*I’m also very young and all this, I feel a lot of pressure of also feeling like we’re living in Octavia Butler Novel and feeling like a lot of doom and gloom, currently where we’re at, with the environment and social political landscape, and disease on and on. So I think that like, this is critical, resistance is critical.*

(Quinn)

*I think there’s also like an aspect I mean, I feel like we are increasingly living in in Octavia Butler Novel and so it feels like this really important skill for the future. I mean for right now, and I feel like it might continue to get worse. It will continue to get worse. And so, I don’t want to be dependent on food that is being trucked in from far away.*

(Danielle)

Another thread that was tied to agency and power is the recognition of some of the impacts of living under a capitalist economic system. Megan shared what that realization was like for her:

*And I think there’s also an aspect of taking yourself out of capitalism when you’re doing it…You’re not part of the pressure of like buying and consuming. It’s really just like, “Okay, well I’m going to see what I can grow.”*

Megan explored growing food as a potential way to decrease her engagement with capitalism. Because she is growing her own food, she does not have to rely as heavily on exchanging money for goods exploitatively produced for profit. Offering another scenario for this interaction between growing food and capitalism, Sarah shared how an economic system can lead to some negative physical impacts:

*I think there is room under a sort of a capitalist system for growing food to also be detrimental, especially when you consider, a lot of the food system is supported by migrant workers and the conditions they work under is like so exploitative. I don’t know, just that negative physical impact.*

The exploitation of migrant workers and other farmworkers is often tied to profit-driven agricultural businesses [73]. Many of these businesses focus more on yields than they do on the health, safety, and wellbeing of their workers. The disregard and exploitation of farmworkers in the United States has deep roots in racism and xenophobia [73]. It is also tied to a culture of production and productivity. Another participant in the White focus groups, Jennifer, acknowledged some of the subtle ways that a culture of productivity convinced her that she should be doing more and growing more:

*I just have…this need to be productive and need to produce and make this garden so big. And I was just going to talk about the negative side of that also, like seeing other people build their gardens much bigger and having capitalism weigh down on us and force us to think that we always need to be productive and doing the most and making the most of our time and our spaces like, “Alright, that was a lot. Let’s dial it back a little bit.”*

Amy offered the following experience in response to Jennifer’s comment:

*Yeah, responding to that…for my work that last year [I] was on a 50-acre production farm and it was motivated by money and yield. And the farm I work at now is less than six acres, technically less than five because one of them is an orchard acre. And the whole approach is different. Yields and efficiency and profit aren’t the main drivers.*

Overall, agency and power showed up in White focus groups as unlearning, critiques of capitalism, and future preparedness. White participants engaged with concepts of resistance, activism, and justice from social positions that differed from Black participants. While people across racial groups talked about topics related to agency and power, Black participants discussed them more frequently. There was also a difference in the emphasis people placed on specific topics. For instance, in one of the Black focus groups, participants dedicated most of the allotted time to talking about activism and resistance. While White participants also named resistance as an impact of urban agriculture, the conversations around resistance were shorter, less emphatic, and did not contain the same sense of urgency as in the Black focus groups. In general, Black focus groups included more co-signing of ideas and intergroup dialogue than the White focus groups.

## 4. Discussion

Rich and interesting information emerged from these data. First, the concept of community care and relationship-building was the number one theme that emerged across all six focus groups. The data also showed that there are both similarities and differences in the perceived impacts of urban agriculture by race. Mentions of spirituality appeared more frequently and more emphatically in the Black focus groups. Both Black and White focus groups emphasized the mental health impacts of urban agriculture. They talked about concepts related to community care and relationship-building as being a primary benefit of growing food. In both groups, people brought up significant issues and barriers around land security.

Many of the emerging themes of the data align with Dr. Monica White’s theoretical framework on collective agency and community resilience, especially within the Black focus groups. Her framework discusses Black agriculture in the context of economic autonomy, commons as praxis, and prefigurative politics. White’s work articulates the centrality of agriculture and Black farmers to Black organizing, self-determination, and liberation [36].

I believe a major strength of this study is that it builds on the quantitative findings of an earlier study that looks at the associations between neighborhood demographics and community food-growing spaces [22]. Through spatial and historical analyses of urban agriculture in Philadelphia, this study found patterning of community gardens and urban farms in Black neighborhoods, low-income neighborhoods, and neighborhoods with low food access. The authors suggested that the establishment of gardens and farms may be a response to the extraction experienced in those neighborhoods. Because that study was nontemporal and noncausal, authors suggest future studies unpack the ways that Black-led agriculture impacts community health. The focus group design employed in this study allowed me to better understand these impacts in a more in-depth way that may not be possible using quantitative methods.

The impacts of urban agriculture on spirituality and collective agency have not been extensively studied. The findings of this focus group study point to some key domains through which agriculture impacts the health of farmers and growers in Philadelphia. Future studies could focus on quantitatively assessing the association between the themes outlined in this article and health and agency. Lastly, all of the outcome variables in this study are not directly observable or measurable. When studying how an exposure affects these kinds of latent concepts, I think the most appropriate way to gain insights and answers is to begin with asking the people impacted directly.

Lastly, this work and the recruitment process highlight the absolute importance of thoroughly engaging with and supporting community partners. As I mentioned earlier, the recruitment goals were met in under two weeks. I attribute this to the deep relationships and connections I have with urban growers and the food justice community throughout the city of Philadelphia. In each of the focus groups, I spent some time asking participants to write down a few topic areas or questions related to urban agriculture that they thought were the most important to include in a survey. The data and findings that emerged from this study directly informed the development of a scale that was recently validated. The goal of this tool is to measure concepts such as spirituality, wellbeing, agency, and power among agriculturalists and agrarian communities.

## Data Availability

The IRB has not granted permission to publicly share the data collected from participants in this study.
